# Vitamin intervention for cytokine storm in the patients with coronavirus disease 2019

**DOI:** 10.1002/mco2.7

**Published:** 2020-06-19

**Authors:** Jian‐Sheng Kang

**Affiliations:** ^1^ Clinical Systems Biology Laboratories The First Affiliated Hospital of Zhengzhou University Zhengzhou China

The coronavirus disease 2019 (COVID‐19) caused by severe acute respiratory syndrome coronavirus 2 (SARS‐CoV‐2) is an ongoing pandemic. According to the data on 18 April 2020, there were more than 2 million people infected by SARS‐CoV‐2 in 211 countries and regions, and the mortality rate of COVID‐19 is about 6.95% (153 406 deaths out of 2 205 733 cases). Two recent clinical summaries of patients with COVID‐19 reported that cytokine storm was associated with the severity and death of patients with COVID‐19.^1^, ^2^

Consequently, it was urgent to seek effective prevention and treatments for the cytokine storm in COVID‐19 patients, especially those under intensive care unit (ICU). The clinical features of 138 patients with COVID‐19 revealed that the number of lymphocytes decreased, the number of monocytes unchanged, and the number of neutrophil significantly increased, which might account for the cytokine storm.^2^ In addition, Guan et al reported that lymphocytopenia was present in 83.2% of 1099 patients with COVID‐19 from 552 hospitals in China.^3^ Tan et al also reported that lymphopenia predicted the severity of COVID‐19.^4^ Overall, these reports suggested that neutrophils rather than lymphocytes might result in cytokine storm.^1^, ^2^, ^3^, ^4^ Normally, neutrophil underwent apoptosis after its activation.^5^ The failure of neutrophil apoptosis might lead to the accumulation of neutrophil and increase the chance of necrosis, which could release the toxic intracellular components and overactivate the immune system.^5^

Interestingly, administration of vitamin C (Vc) could decrease the number of neutrophil in septic animals, and Vc‐deficient neutrophils were not efficiently removed by macrophages.^5^ In addition, because the caspase‐dependent apoptosis was oxidant sensitive, Vc was expected to escort the apoptotic process and avoid inflammatory necrosis following the activation of neutrophil.^5^ Moreover, neutrophils need millimolar concentrations of intracellular Vc for their pathogen‐eradicating functions and motility.^5^ Consequently, the oral administration of sufficient Vc might be sufficient to ameliorate the cytokine storm caused by neutrophils. Interestingly, a clinical trial of high‐dose Vc infusion for COVID‐19 was registered (ClinicalTrials: NCT04264533) on 11 February 2020.

Th17, an interleukin‐17 (IL‐17) secreting CD4^+^ T cell, has a unique link to augment and coordinate the functions of neutrophils.^6^ Th17 was significantly increased in the ICU patients with COVID‐19.^1^ IL‐6 is one of critical factors for the differentiation and development of Th17 cell.^6^ Tocilizumab could bind and block the effects of IL‐6 and might have a potential effect for COVID‐19 treatment, so that a clinical trial was also registered (ChiCTR2000029765) on 13 February 2020. Importantly, 1‐10 nM of vitamin D (Vd) via vitamin D receptor (VDR) could efficiently suppress the cytokine production of Th17 by inducing the expression of C/EBP homologous protein (CHOP).^7^ Together, the simultaneous administration of sufficient Vc and Vd might ameliorate the neutrophil‐related cytokine storm in the patients with COVID‐19, especially those under ICU (Figure 1).

**FIGURE 1:**
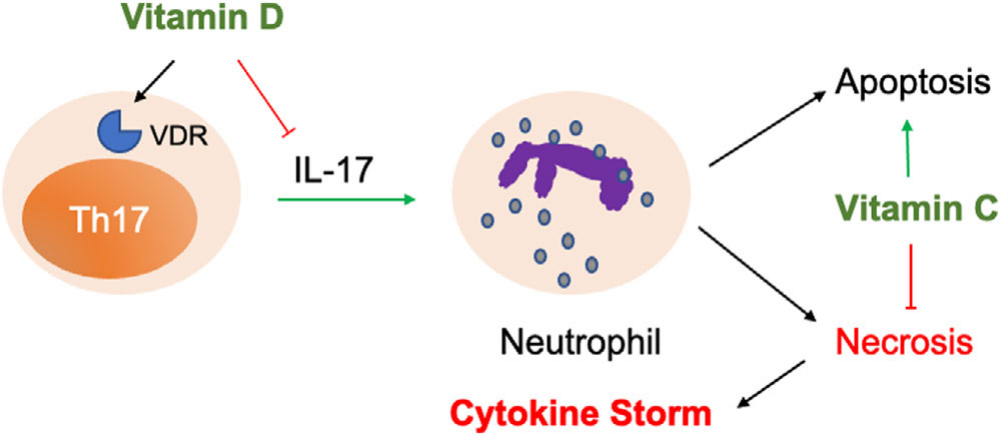
Vitamin Intervention for neutrophil‐related cytokine storm. Neutrophil‐related cytokine storm was reported to be associated with the severity and death of patients with COVID‐19. Th17, an IL‐17 secreting CD4^+^ T cell, has a unique link to augment the function of neutrophil. Vitamin D via vitamin D receptor (VDR) could efficiently suppress the cytokine production of Th17. Vitamin C could decrease the number of neutrophils, escort the apoptotic process, and avoid inflammatory necrosis following the activation of neutrophil

Recommended dietary allowances (RDAs) for Vd are as follows: 800 IU for a level of 20 ng/mL (50 nM) and 1600 IU for a level of 30 ng/mL (75 nM).^8^ These nanomolar concentrations of Vd should sufficiently induce CHOP expression via VDR to inhibit the cytokine production of Th17 and the Th17‐related augmentation of neutrophils.^7^ Based on the many mechanisms of Vd reducing the risk of viral infections, Grant et al recommended that a daily dose (10 000 IU) of Vd should be taken to achieve an optimal level of 40‐60 ng/mL (100‐150 nM), and claimed without adverse effects.^9^ However, a recent preprint of medRxiv about Vd suppressing cytokine storm in COVID‐19 patients was withdrawn by the authors because they noticed the idiosyncrasy of Vd across different countries.^10^ In general, as for the prevention or treatments of the neutrophil‐related cytokine storm, to meet the requirements of 50% of the population, a 400‐600 IU daily dose of Vd should be safe and sufficient, whereas the RDA of Vd (800‐1600 IU daily) could meet the needs of 97.5% of individuals.^8^

Considering that Vc could hinder the function of interferon‐gamma^11^ and that leukocytes including neutrophils and monocytes accumulate maximal Vc concentrations at dietary intakes of ∼100 mg/day,^5^ it is important to use safe dose for the intervention of neutrophil‐related cytokine storm and avoid slowing the clearance of viruses by immune response. RDA for Vc is about 90 mg daily, interestingly, which was selected at 80% saturation of neutrophils.^12^ Levin et al suggested that the safe dose of Vc was less than 1000 mg daily.^13^ Wang et al recently suggested that Vc and Vitamin E (Ve) might ameliorate cardiac injuries of COVID‐19 patients as an antioxidant.^14^ Compared to other antioxidants and water‐insoluble Ve, Vc and Vd are preferred natural substrates^15^ and modulators of neutrophil function and fate. In brief, an oral daily dose of Vc between 90 and 1000 mg might avoid inhibiting innate immunity and safeguard the homeostasis of neutrophils.

On the other hand, Cheng discussed that Vc up to 1.5 g/kg body weight might be safe and without major adverse events^16^, and cited that high‐dose intravenous Vc (50‐200 mg/kg body weight) was recommended in the expert consensus statement on comprehensive treatment of COVID‐19 from Shanghai, China.^17^ The high‐dose intravenous Vc might act as a pro‐oxidant in immune cells and induce glyceraldehyde‐3‐phosphate dehydrogenase inhibition and related immunosuppression via NAD^+^ (nicotinamide adenine dinucleotide) depletion.^18^ Due to the roles of high‐dose Vc in immunosuppression, the dosage of Vc should be deliberated on the details to take into account the delay of virus clearance.

This perspective focused on the aberration of neutrophil as one of the clinical characteristics of COVID‐19.^1^, ^2^, ^3^ Consequently, the dose ranges of Vc and Vd among 90‐1000 mg and 400‐1600 IU, respectively, might be enough and safe for the prevention or treatment of the cytokine storm caused by neutrophil, whereas for the cases not related to neutrophil, high dose of Vc or Vd might be also helpful for the treatment of COVID‐19 patients due to the multifaceted roles of Vd and Vc.^9^, ^16^, ^18^ As the global pandemic is still growing, if the vitamin intervention for cytokine storm could be proved to be applicable and efficient, it would be tremendously helpful and easy to protect the patients with COVID‐19 worldwide from the cytokine storm‐related death.

## CONFLICT OF INTEREST

The author declares no conflict of interest.
